# Residual Tumor Volume as Best Outcome Predictor in Low Grade Glioma – A Nine-Years Near-Randomized Survey of Surgery vs. Biopsy

**DOI:** 10.1038/srep32286

**Published:** 2016-08-30

**Authors:** Roland Roelz, David Strohmaier, Ramazan Jabbarli, Rainer Kraeutle, Karl Egger, Volker A. Coenen, Astrid Weyerbrock, Peter C. Reinacher

**Affiliations:** 1 Department of Neurosurgery, Medical Center – University of Freiburg, Breisacher Str. 64, 79106 Freiburg, Germany; 2 Department of Stereotactic and Functional Neurosurgery, Medical Center – University of Freiburg, Breisacher Str. 64, 79106. Freiburg, Germany; 3 Department of Neurosurgery, University Hospital Essen, Hufeland str. 55, 45147, Germany; 4 Department of Nursing-IT, Medical Center – University of Freiburg, Hugstetter Str. 55, 79106, Freiburg, Germany; 5 Department of Neuroradiology, Medical Center – University of Freiburg, Breisacher Str. 64, 79106 Freiburg, Germany

## Abstract

Diffuse low grade gliomas (DLGG) are continuously progressive primary brain neoplasms that lead to neurological deficits and death. Treatment strategies are controversial. Randomized trials establishing the prognostic value of surgery do not exist. Here, we report the results of a nine-year near-randomized patient distribution between resection and biopsy. Until 2012, the Department of Neurosurgery and the Department of Stereotactic Neurosurgery at the University Medical Center Freiburg were organized as separate administrative units both coordinating DLGG patient treatment independently. All consecutive adult patients with a new diagnosis of DLGG by either stereotactic biopsy or resection were included. Pre- and post-operative tumor volumetry was performed. 126 patients, 87 men (69%), 39 women (31%), median age 41 years, were included. 77 (61%) were initially managed by biopsy, 49 (39%) by resection. A significant survival benefit was found for patients with an initial management by resection (5-year OS 82% vs. 54%). The survival benefit of patients with initial resection was reserved to patients with a residual tumor volume of less than 15 cm^3^. Maximum safe resection is the first therapy of choice in DLGG patients if a near-complete tumor removal can be achieved. Accurate prediction of the extent-of-resection is required for selection of surgical candidates.

Diffuse supratentorial low grade glioma in adults (DLGG) is a heterogenous neuroectodermal tumor entity accounting for about 15% of all glial brain neoplasms^1^. It is a chronic progressive disease of the central nervous system that, by infiltrative growth and malignant transformation, leads to neurological deficits and death. Since class I evidence for the benefit of any medical treatment does not exist, optimal management of DLGG is still controversial^2^,^3^,^4^,^5^,^6^,^7^,^8^,^9^. An increasing body of evidence suggests, that an early surgical intervention is superior compared to a “watch and wait” strategy^8^,^10^.

Different surgical strategies have been tried including stereotactic biopsy followed by brachytherapy, chemotherapy or external radiation or primary resection.

To date, no randomized controlled trial comparing surgical resection with biopsy has been conducted in patients with DLGG^11^. Given the growing evidence of the benefits of an early surgical intervention, it is unlikely that such a trial will ever be performed^12^,^13^. The closest approach to a patient randomization between biopsy and surgery originates from a retrospective population-based study comparing two Norwegian hospitals with different management strategies^14^. Herein, a significant survival benefit (74% vs. 60% 5-year OS, 68% vs. 44% 7-year OS) was found if the principal initial management strategy of patients with DLGG was surgery. Consequently and in line with present international guidelines^8^, surgical resection to the maximum safe extent at present is the suggested first-line therapy for DLGG.

A starting position comparable to that described by Jakola *et al.* historically existed at our center. Until 2012, patients with a first diagnosis of DLGG were treated and followed either by the department of Stereotactic Neurosurgery or the Department of Neurosurgery – both acting independently - at our university medical center. This situation led to a near-randomized distribution of patients with DLGG to initial management either by biopsy or surgical resection.

In the present study, the results of an early resective intervention are compared to a management with biopsy alone with special emphasis on a pre- and post-operative tumor volumetry.

## Materials and Methods

The study design and methods were approved by the Ethics Committee of the University Medical Center Freiburg. The methods were carried out in accordance with the approved guidelines. The trial was registered with the German Clinical Trial Registry (DRKS, Unique identifier: DRKS00009264). All persons or their relatives gave their informed consent within written treatment contract on admission and therefore prior to their inclusion in the study.

### Clinical Setting

Until 2012, the Department of Neurosurgery and the Department of Stereotactic Neurosurgery at the University Medical Center Freiburg were organized as two separate administrative units. Both Departments acted independently in terms of patient care. Patients with DLGG were allocated to either department by a near-randomized process resulting from random referral. Both departments followed their patients in separate outpatient clinics and independently decided on choices of adjuvant treatment strategies. The mean distance between the patients’ place of residence at diagnosis and our medical center was calculated using Google Maps (http://maps.google.com/maps, access: 01/2016) to test for comparability of the catchment area. Stereotactic and surgical samples were equally analyzed by the Department of Neuropathology, Medical Center – University of Freiburg.

### Eligibility

All patients 18 years or older with a first histological diagnosis of a supratentorial DLGG (WHO°II) from either a stereotactic biopsy or a neurosurgical resection between 2004 and 2012 in the Department of Neurosurgery or the Department of Stereotactic and Functional Neurosurgery of the Medical Center – University of Freiburg, were included in the study. Patients with evidence of a gemistocytic histology were excluded due to the known aggressive clinical behavior of gemistocytic gliomas^15^,^16^. Patients with gliomatosis cerebri (defined as evidence of initial tumor infiltration of 3 or more lobes) were excluded.

### Variables

Patient and treatment characteristics were retrieved from medical records (primary source). If patients received a surgical resection within 3 months after biopsy the initial management was classified as “resection”. Pre-operative MRI were available in 118 of 126 patients (94%) [FLAIR/T2w in 100 (79%), T1w in 18 (14%)]. Pre-operative tumor volumetry was based on computed tomography (CT) in the remaining 8 patients (6%). Contrast-enhanced pre-operative T1w MRI were available in 114 patients (90%). At least one follow-up MRI obtained within 6 months after resection was available in 45 patients (92% of patients with an initial resection). Post-operative tumor volumetry was based on CT in 4 patients (8%). Pre- and post-operative tumor volumetry was manually performed using the open source software OSIRIX (http://www.osirix-viewer.com).

Presence and severity of comorbidities were evaluated by the Charlson Comorbidity Index^17^. The Karnofsky Performance Score (KPS) was used to determine the functional status of the patients upon study inclusion. The items of the Pignatti-Score^18^ (i.e. age ≥ 40 years, tumor diameter ≥ 6 cm, tumor crossing midline, presence of neurological deficit, astrocytic histology) were included in the statistical analysis. Tumor location, initial contrast enhancement, maximum tumor diameter and eloquence were independently reviewed by two neurosurgeons (P.R., R.R.). Eloquence was graded according to the Sawaya-Score^19^. Post-interventional neurological deficits were dichotomized in transient (<6 months) or permanent (>6 months) deficits.

### Follow-up and Statistical Analysis

Overall survival (OS) was the primary end point of the study. Survival was calculated from the date of the first histological diagnosis. Patients were followed until death or September 30, 2015. Six patients (5%) were lost to follow-up. They were censored at the date of the recorded last contact.

Univariate statistics were performed by log-rank test, Fisher exact test, Pearson χ^2^ test, Mann-Whitney-U test or one-way ANOVA (Kruskal-Wallis test with Dunns multiple comparison test), as appropriate. To determine independent predictors of patient survival significant variables from the initial univariate analyses were included in two multivariable cox-regression models. All patients were included in the first model. The second model assessed only patients with initial resection and included the new variable residual tumor volume (RTV). Insignificant variables were excluded by backward elimination in both models. The Pignatti-Score was excluded from the multivariable analysis despite being a significant variable for OS in univariate statistics as it represents a compound variable. All reported p-values were two sided, and p < 0.05 was considered statistically significant. All statistical analyses were performed using GraphPad Prism version 5 (GraphPad Software, San Diego, USA) or Stata 13 statistical software (StataCorp. 2013. Stata Statistical Software: Release 13. College Station, TX, USA).

## Results

### Biopsy vs. Resection, Near-Randomisation Between Two Departments

Between 2004 and 2012, 126 patients with a first histological diagnosis of DLGG (excluding gemistocytic gliomas and gliomatosis cerebri) were surgically treated at the University Medical Center Freiburg. 77 patients were managed by the Department of Stereotactic Neurosurgery (hereafter referred to as “Biopsy” group) and initial management was stereotactic biopsy. 49 patients underwent an early surgical resection and were treated at the Department of Neurosurgery (hereafter referred to as “Resection” group). 3 patients received an initial biopsy and were resected within 3 months. These patients were included in the “Resection” group. The postal codes of patients at the date of diagnosis were available for 47 of 49 patients (96%) with initial resection and 75 of 77 (97%) with initial biopsy. The catchment area (i.e. the mean distance between the patients’ place of residence at diagnosis and our medical center) was comparable for both departments: Patients in the “Resection” group lived a mean 84.6 (±123.6) and patients in the “Biopsy” group a mean 90.9 (±104.4) kilometres from our centre (p = 0.36).

**Figure 1:**
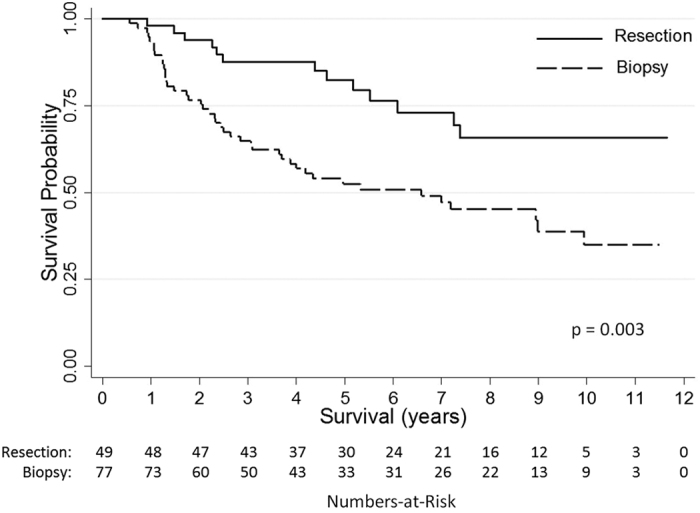
Overall survival of patients with a first diagnosis of low grade glioma and initial management by either resection or biopsy.

A dichotomized overview comparing patient, tumor and treatment characteristics of both patient cohorts is summarized in Table 1. Both groups were comparable for most variables. However, significant differences were found for the year of diagnosis (“Biopsy”: 2006 vs. “Resection”: 2008). The “Biopsy” cohort contained significantly more patients with an astrocytoma histology (57 vs 37%). The mean tumor volume was significantly larger (54 vs 75 cm^3^) in the “Resection” group. Biopsy patients were more likely to receive early radiotherapy (33 vs. 4%) or chemotherapy (29 vs. 10%). The median follow-up of “Biopsy” patients was 4.3 (IQR: 2.1–8.2) years and 5.6 (IQR: 4.0–8.9) years for “Resection” patients. By the end of the observation period, significantly more “Biopsy” patients had died (56% vs. 27%). Median OS was 6.7 years in the “Biopsy” group and not reached in the “Resection” group (Fig. 1). OS after 5 years was 82% for “Resection” patients compared to 54% with initial biopsy and 67% vs. 38% after 10 years. There was no surgical mortality. No post-operative deficits occurred in the “Biopsy” cohort. 7 patients (14%) with an initial management by resection developed a new post-operative deficit which was permanent (i.e. persisting >6 months) in 4 (8%).

Both departments pursued different policies regarding the timing of adjuvant therapy. Patients initially managed by biopsy were more frequently referred to an early (within 6 months after diagnosis) radiotherapy (33% vs. 8%) or chemotherapy (30 vs. 11%) if the initial histological diagnosis was Astrocytoma (Supplementary Figure 1 and Table 1, see Adjuvant Therapy).

A new or worsened post-operative deficit was observed in 7 “Resection” patients (14%). Recovery was observed in 3 of these and 4 patients (8%) remained with a permanent deficit. No permanent deficits were observed among patients initially treated by biopsy.

### Prognostic Factors for Overall Survival

In order to establish prognostic factors for a longer OS, patient data from both departments were pooled (Table 2). Patient, tumor and treatment characteristics that had a significant negative impact on OS in univariate analyses were **higher age at diagnosis**, higher **Pignatti-Score**, **astrocytoma histology**, a tumor location in the **parietal** lobe or **otherwise not specified** location (other, i.e.: 7 thalamic, 4 bilobar, 2 occipital, 1 basal ganglia), and **initial management with biopsy**. Early radiotherapy was also associated with a shorter OS. However, this treatment strategy was predominantly followed in patients with initial management by biopsy and astrocytoma histology, both being established risk factors for shorter OS (Supplementary Figure 1). “Early radiotherapy” was therefore excluded as a variable from further statistical analyses.

22 patients (29%) with an initial management with biopsy underwent a surgical resection after a mean time of 2.8 (±2.4) years. This, however, had no effect on OS. The median OS of patients who crossed over to a resection after an initial management by biopsy was 7.8 years vs. 5.0 years in patients who never underwent a resection (p = 0.67).

In a multivariable Cox-regression model statistical significance for OS was maintained for **age at diagnosis** (HR 1.03 per year), **astrocytoma histology** (HR 2.25) and **otherwise not specified tumor location** (HR 3.3).

Remarkably, the important impact of the initial management strategy (biopsy vs. resection) on OS (Fig. 1) was not maintained in the multivariable model. A comparison of both groups (“Resection” vs. “Biopsy”) regarding each histological subtype did not show significant differences of OS (Supplementary Figure 2). Consequently, factors other than the initial management strategy were essential determinants for survival differences between patients initially managed by biopsy or resection.

**Figure 2:**
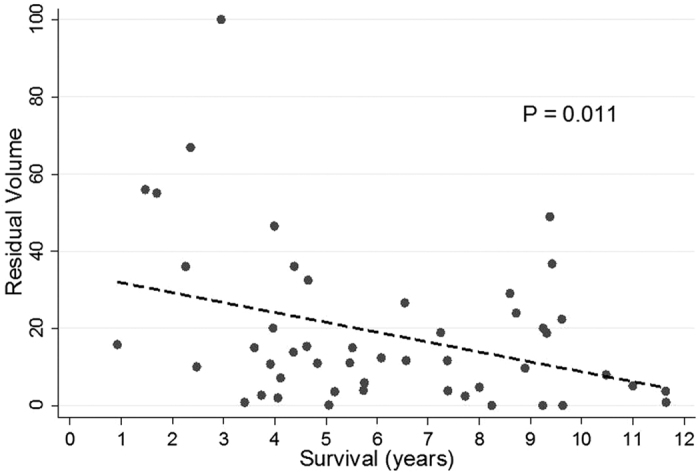
Correlation between overall survival and residual tumor volume.

We therefore evaluated the **residual tumor volume** (**RTV**) in patients with an initial management by resection. A significant correlation between better OS and smaller RTV was found (Fig. 2). A dichotomization of these patients was performed according to the RTV (RTV < 15 cm^3^, RTV > 15 cm^3^).

A multivariable Cox-regression model to assess significant factors for OS was applied to the patients with initial resection including the RTV (Table 3). Herein, **RTV** > **15** cm^3^ (HR 3.8) and **astrocytoma histology** (HR 5.4) were predictors of shorter survival. Age at diagnosis was not associated with inferior OS.

**Figure 3:**
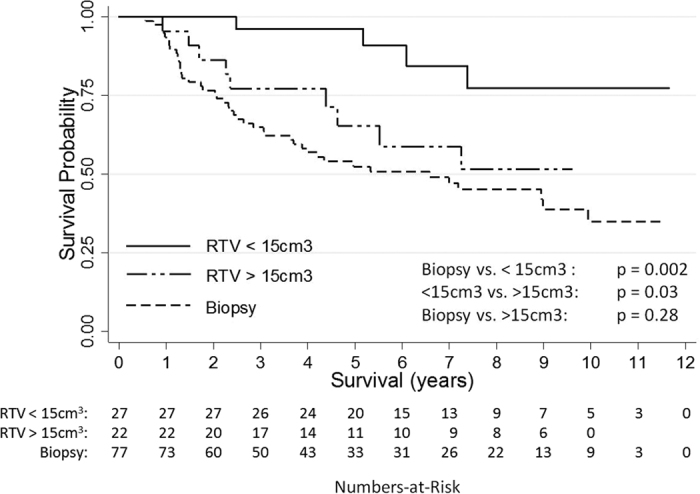
Overall survival of low grade glioma patients according to the residual tumor volume (RTV). Overall survival of patients with a RTV of more than 15 cm^3^ did not have an improved prognosis compared to patients with an initial management by biopsy.

Based on this analysis the patients were re-grouped into three prognostically relevant Residual Tumor Volume groups (RTV < 15 cm^3^, >15 cm^3^ and Biopsy), (Supplementary Table 1). 21 of 27 patients (78%) with a RTV < 15 cm^3^ were alive at the end of follow up. The 5 and 10-year OS rate in this group was 82 and 69%, respectively. In comparison, OS did not differ significantly between patients managed by either biopsy or resection with a RTV > 15 cm^3^ (6.7 vs. 10.6 years median OS). 43 patients (56%) initially managed by biopsy and 12 patients (55%) with a RTV > 15 cm^3^ were dead by the end of follow-up (Fig. 3).

There was no surgical mortality. New post-operative deficits occurred in 5 patients from the RTV < 15 cm^3^ group (19%) and 2 patients from the RTV > 15 cm^3^ (9%). Permanent deficits were observed in 2 patients from both surgical groups (7% and 9%, respectively).

Notably, a large heterogeneity was found for patient, tumor and treatment characteristics between these three groups rendering a comparability questionable. Most impressively, the tumor volume of patients with a RTV > 15 cm^3^ was significantly bigger than of the “Biopsy” and RTV < 15 cm^3^ group. In order to answer the question whether the comparably poor OS of the RTV > 15 cm^3^ group was a result of selection bias, a group matching the characteristics of the RTV > 15 cm^3^ group was extracted from the “Biopsy” patients (Supplementary Table 2). No relevant difference in OS (9.1 vs 10.6 years) was observed between these groups (Supplementary Figure 3).

## Discussion

In agreement with the closest approach to a randomization of patients with DLGG between surgery and biopsy reported by Jakola *et al.* survival analysis of patients initially managed by either biopsy or resection revealed a significantly better OS for patients managed by an early surgical intervention. The 5-year OS rate was 82% for “Resection” patients compared to 54% for “Biopsy” patients (67% vs. 38% after 10 years). We hypothesize that the survival benefit of the surgical group is even underestimated as patients initially managed by biopsy were more likely to receive an early adjuvant treatment (53% vs. 14%). A later surgical intervention after initial management by biopsy (as performed in 22 patients initially managed by biopsy after a mean 2.8 years) did not have an impact on OS corroborating the importance of the initial treatment choice.

A more differentiated analysis of our patient data, especially with respect to the pre- and post-operative tumor volume revealed however, that the OS benefit was exclusively reserved to patients in whom a resection with a RTV of less than 15 cm^3^ was achieved. While these patients had a good prognosis (77% 10-year OS) patients with a RTV of more than 15 cm^3^ did not fare better than patients managed by biopsy alone. This effect was maintained when the RTV > 15 cm^3^ cohort was compared to a subset of “Biopsy” patients that was matched for known prognostic factors.

In line with the pertinent literature but in contrast to the series of Jakola *et al.* initial management by stereotactic biopsy in our study was not associated with surgical morbidity^20^.

Interestingly, tumor size per se, previously validated as a strong prognostic factor in large patient series^18^,^21^, is not prognostic in a near-randomized setting between surgery and biopsy. We suggest that this finding further supports the prognostic value of an early surgical intervention, since a poor starting position provided by tumor characteristics (evaluated here: tumor size) can possibly be reversed by an aggressive surgical therapy.

In large patient cohorts, astrocytoma histology was associated with an inferior prognosis compared to mixed gliomas and oligodendrogliomas^8^,^22^,^23^. In the present study, a pure astrocytoma histology was significantly more frequently diagnosed in specimen obtained by biopsy compared to surgery (57% vs. 37%) potentially indicating a selection bias of prognostically poor patients to biopsy. We suggest, however, that this represents a sampling error due to the known limited representation of histological tumor features in biopsy specimen^24^,^25^,^26^,^27^. Per se, there is no unequivocal association between histological and molecular features and resectability of DLGG^22^,^23^. Further, the frequency of astrocytoma histology was balanced between patients with a RTV < 15 cm^3^ and RTV > 15 cm^3^ supporting this notion.

The precise delineation of a beneficial extent of resection or residual tumor volume remains a matter of debate. While the largest investigation addressing this aspect by Capelle *et al.* suggested a residual tumor volume of less than 10 cm^3^ to be the crucial factor for a good prognosis, in particular due to a delay of anaplastic transformation^21^, others have suggested different percentages of tumor removal/residual tumor that proved valuable^28^,^29^,^30^,^31^,^32^,^33^. In light of the present study, we clearly challenge the notion, that any surgical intervention improves patient survival in DLGG. Instead, a differentiated and personalized view is necessary for appropriate treatment decisions.

Form our data we would recommend performing surgery in patients if a resection with optimally none tumor residual or at least a residual volume in the range of up to a maximum of 15 cm^3^ can be achieved at an acceptable risk. In cases, where this maximal residual tumor volume is likely to be exceeded, a preferred strategy could be (stereotactic) biopsy followed by adjuvant therapy bearing a neoadjuvant approach with a resection after tumor volume reduction by chemotherapy in mind^34^,^35^.

Taken together, the data summarized here underscore the fact that most DLGG cannot be considered benign lesions unless a significant tumor volume reduction can be achieved. Adjuvant therapy is indicated in patients with poorly resectable DLGG. Furthermore, neurosurgeons are encouraged to continue the quest for an increased resectability of DLGG by improving surgical techniques while preserving function and quality of life at the same time^29^. Prediction of the attainable RTV and risk stratification at the individual level (surgeon and patient) needs to be refined to ameliorate treatment decisions^36^,^37^,^38^.

### Limitations of the study

Our study is subject to the common constraints of a retrospective investigation. We limited the study to patients with a first diagnosis of DLGG. A histological sampling error cannot be ruled out as the diagnostic accuracy of biopsy specimen may be variable^24^,^25^,^26^,^27^. The greatest possible conformity of histological analyses is provided by the fact that all specimen were analyzed by the same neuropathology unit. Due to the retrospective nature of this study that included patients with a first diagnosis as early as 2004 molecular tumor characteristics were only available in part and therefore not included into the present analysis. A certain treatment selection bias has to be acknowledged as patients harboring larger tumors were more likely to receive a resection (mean tumor volume, “Resection”: 75 mm^3^ “Biopsy”: 54 mm^3^). In turn, patients with tumors within the basal ganglia and thalamus were more likely to be managed by biopsy. We did not exclude these patients as the a priori intention of the present study was to investigate the near-randomized patient distribution between a management by initial surgery or biopsy irrespective of tumor size or location. Patients initially managed by biopsy were more likely to receive early adjuvant therapy creating a certain heterogeneity of both groups.

## Conclusion

According to our study, the treatment option of choice for patients with a first diagnosis of DLGG is a resection to the maximum possible extent. If a complete or near-complete tumor volume reduction is not feasible, surgery does not provide a survival benefit but exposes patients to the risk of neurological injury. In these cases, biopsy should be the initial management. Potentially, neoadjuvant chemotherapy may render tumors safely operable to a prognostically valuable extent. Further investigations are needed to precisely predict the attainable RTV to improve selection of surgical candidates.

## Additional Information

**How to cite this article**: Roelz, R. *et al.* Residual Tumor Volume as Best Outcome Predictor in Low Grade Glioma – A Nine-Years Near-Randomized Survey of Surgery vs. Biopsy. *Sci. Rep.* **6**, 32286; doi: 10.1038/srep32286 (2016).

## Supplementary Material

Supplementary Information

## Figures and Tables

**Table 1:** Comparison of Patient, Tumor and Treatment Characteristics in Both Departments.

	Initial Management		Univariate Statistics
	Biopsy	Resection	
Patient characteristics			
Number of patients	77	49	
Year of first diagnosis, (IQR), y	**2006** (**2005–2008**)	**2008** (**2006–2011**)	**p = 0**.**0025**
Sex			p = 0.24
Male	50 (65%)	37 (76%)	
Female	27 (35%	12 (25%)	
Age at diagnosis, median (IQR), y	44 (35–60)	39 (33–46)	p = 0.096
Follow-up, median, (IQR), y	4.3 (2.1–8.2)	5.6 (4.0–8.9)	P = 0.066
Status			χ^2^ (2, N = 126) = 10.4 **p = 0**.**005**
Dead	**43** (**56%**)	**13** (**27%**)	
Alive	31 (40%)	33 (67%)	
Unknown	3 (4%)	3 (6%)	
Overall Survival, median, y	6.7	Not reached	**p = 0**.**003**
5-year survival rate (%)	54%	82%	
10-year survival rate (%)	38%	69%	
Preoperative KPS, mean (SD), %	90 (8.1)	92 (8.6)	p = 0.08
Charlson comorbidity index, mean (SD)	0.32 (1.1)	0.37 (0.8)	p = 0.19
Initial symptoms			χ^2^ (3, N = 126) = 0.45 p = 0.93
None (incidental)	5 (6%)	2 (4%)	
Seizure	49 (64%)	34 (69%)	
Headache	10 (13%)	6 (12%)	
Neurological deficit	23 (30%)	15 (31%)	
Pignatti score, mean (SD)	1.8 (1.0)	1.7 (1.3)	p = 0.39
Tumor Characteristics			
Histopathology			χ^2^ (2, N = 126) = 8.5 **p = 0**.**014**
Astrocytoma WHO°II	**44** (**57%**)	**18** (**37%**)	
Oligoastrocytoma WHO°II	27 (35%)	19 (39%)	
Oligodendroglioma WHO°II	6 (8%)	12 (24%)	
Tumor size			
Maximum diameter, mean, (SD), mm	50 (19)	58 (23)	p = 0.051
Tumor volume, mean, (SD), mm^3^	**54** (**53**)	**75** (**61**)	**p = 0**.**045**
Eloquent location (Sawaya-Score)			χ^2^ (2, N = 126) = 3.2 p = 0.20
1	5 (6%)	8 (16%)	
2	46 (60%)	25 (51%)	
3	26 (34%)	16 (33%)	
Bilateral tumor extension	4 (5%)	3 (6%)	p = 1.00
Left Hemisphere	45 (58%)	25 (51%)	p = 0.45
Preoperative contrast enhancement	14/73§ (19%)	13/41§ (32%)	p = 0.17
Location of tumor			χ^2^ (4, N = 126) = 5.0 p = 0.29
Frontal	29 (38%)	22 (45%)	
Temporal	10 (13%)	11 (22%)	
Parietal	7 (9%)	2 (4%)	
Insula	20 (26%)	11 (22%)	
Other	11 (14%)	3 (6%)	
Surgical Characteristics			
Number of tumor resections			
0 (Biopsy only)	55 (71%)	—	
1	13 (17%)	25 (51%)	χ^2^ (2, N = ) = 2.2 p = 0.34
2	6 (8%)	21 (43%)	χ^2^ (2, N = ) = 2.2 p = 0.34
>2	3 (4%)	3 (6%)	
Post operative deficits†	0 (0%)	7 (14%)	**p = 0**.**001**
Post-operative deficits >6 months	0 (0%)	4 (8%)	**p = 0**.**02**
			χ^2^ (4, N = ) = 13.2 **p = 0**.**011**
Adjuvant Therapy	Available: 66 (86%)	Available: 49 (100%)	χ^2^ (4, N = ) = 14.9 **p = 0**.**005**
None	22 (33%)	23 (47%)	
Early radiotherapy	**22** (**33%**)	**2** (**4%**)	
Ever radiotherapy	37 (56%)	21 (43%)	
Early chemotherapy	**19** (**29%**)	**5** (**10%**)	
Ever chemotherapy	41 (62%)	23 (47%)	

^§^Number of available pre-operative contrast-enhanced MRI.

^†^Deficits after first intervention (biopsy or 1^st^ surgery), permanent deficit was defined as a new deficit persisting >6 months.

**Table 2:** Multivariate Analysis of Prognostic Factors.

Overall Survival Analysis Failure Event: Death	LGG Patients	Univariate Statistics	Multivariate Cox-Regression
Patient characteristics			
Number of patients	126		
Year of first diagnosis, (IQR), y	2007 (2005–2010)		
Sex		p = 0.71	
Male	87 (69%)		
Female	39 (31%)		
Age at diagnosis, median (IQR), y	41 (35–51)	**p = 0**.**001**	**p = 0**.**001** (95% CI: 1.01–1.05) **HR: 1**.**03** (**per year**)
Follow-up, median (IQR), y	4.9 (2.5–8.4)		
Status			
Dead	56 (44%)		
Alive	64 (51%)		
Lost to follow-up	6 (5%)		
Overall Survival, median, y	9.1		
5-year survival rate (%)	65%		
10-year survival rate (%)	49%		
Preoperative KPS, mean (SD), %	90.6 (8.3)	p = 0.12	
Charlson comorbidity index, mean (SD)	0.34 (1.0)	p = 0.76	
Initial symptoms			
None (incidental)	7 (6%)	p = 0.61	
Seizure	83 (66%)	p = 0.61	
Headache	16 (13%)	p = 0.73	
Neurological deficit	38 (30%)	p = 0.22	
Pignatti score, mean (SD)	1.7 (1.1)	**p** < **0**.**001**	
Tumor Characteristics			
Histopathology			
Oligodendroglioma WHO°II	18 (14%)	Reference	Reference
Oligoastrocytoma WHO°II	46 (37%)	p = 0.098	
Astrocytoma WHO°II	62 (49%)	**p = 0**.**002**	**p = 0**.**010** (95% CI: 1.3–4.2) **HR: 2**.**25**
Tumor size			
Maximum diameter, mean, (SD), mm	53 (21)	p = 0.85	
Tumor volume, mean, (SD), mm^3^	62 (57)	p = 0.69	
Eloquent location (Sawaya-Score)		p = 0.066	
1	13 (10%)		
2	81 (64%)		
3	42 (33%)		
Bilateral tumor extension	7 (6%)	p = 0.29	
Left Hemisphere	70 (56%)	p = 0.40	
Preoperative contrast enhancement	27/114§ (24%)	p = 0.71	
Location of tumor			
Frontal	51 (40%)	Reference	Reference
Temporal	21 (17%)	p = 0.78	p = 0.69
Parietal	9 (7%)	**p = 0**.**006**	p = 0.05
Insula	31 (25%)	p = 0.21	p = 0.64
Other	14 (11%)	**p** < **0**.**001**	**p = 0**.**007** (95% CI: 1.4–7.9) **HR: 3**.**3**
Initial management by biopsy	77 (61%)	**p = 0**.**0018**	p = 0.083
Resection after initial management by biopsy	22 (29%)	p = 0.67	
Adjuvant Therapy	Available: 115 (91%)		
None	45 (39%)	p = 0.18	
Early radiotherapy	25 (22%)	**p** < **0**.**001**	
Ever radiotherapy	58 (50%)	p = 0.11	
Early chemotherapy	24 (21%)	p = 0.91	
Ever chemotherapy	64 (56%)	p = 0.51	

^§^Number of available pre-operative contrast-enhanced MRI.

**Table 3:** Multivariate Analysis of Prognostic Factors in Patients with Initial Resection.

Overall Survival Analysis Failure Event: Death	LGG Patients Resection	Multivariate Cox Regression
Number of patients	49	
Age at diagnosis, median (IQR), y	39 (33–46)	p = 0.71
Histopathology		
Oligodendroglioma WHO°II	12 (24%)	Reference
Oligoastrocytoma WHO°II	19 (39%)	
Astrocytoma WHO°II	18 (37%)	**p = 0**.**005** (95% CI: 1.7–17.8) **HR: 5**.**4**
Residual Tumor Volume		
Residual tumor volume <15 cm^3^	27 (55%)	Reference
Residual tumor volume >15 cm^3^	22 (45%)	**p = 0**.**034** (95% CI: 1.1–13.0) **HR: 3**.**8**
