# Supplementary material for: Residual Tumor Volume as Best Outcome Predictor in Low Grade Glioma – A Nine-Years Near-Randomized Survey of Surgery vs. Biopsy
Source: Sci Rep. 2016 Aug 30;6:32286. doi: 10.1038/srep32286 (PMC5004168; doi:10.1038/srep32286)
Supplement: Supplementary Information [file srep32286-s1.pdf]

Supplementary Material

Residual Tumor Volume as Best Outcome Predictor in Low Grade Glioma – A Nine-Years Near-Randomized Survey of Surgery vs. Biopsy

Roland Roelz[1,2,*], David Strohmaier[2], Ramazan Jabbarli[1,3], Rainer Kraeutle[4], Karl Egger[5], Volker A. Coenen[2], Astrid Weyerbrock[1], Peter C. Reinacher[2]

[1]Department of Neurosurgery, Medical Center – University of Freiburg, Breisacher Str. 64, 79106 Freiburg, Germany (RR, DS, AW, MM)

[2]Department of Stereotactic and Functional Neurosurgery, Medical Center – University of Freiburg, Breisacher Str. 64, 79106 Freiburg (PCR, VAC)

[3]Department of Neurosurgery, University Hospital Essen, Hufelandstr. 55, 45147, Germany (RJ)

[4]Department of Nursing-IT, Medical Center – University of Freiburg, Hugstetter Str. 55, 79106 Freiburg (RK)

[5]Department of Neuroradiology, Medical Center – University of Freiburg, Breisacher Str. 64, 79106 Freiburg, Germany (KE)

***Corresponding Author:**

Roland Roelz, MD

Department of Neurosurgery, University Medical Center Freiburg

Breisacher Strasse 64, 79106 Freiburg, Germany

e-mail: roland.roelz@uniklinik-freiburg.de

Phone: 0049 – 761 270 50010, Fax: 0049 – 761 270 93090

Running Title:

Near-Randomization of Surgery vs. Biopsy in Low Grade Glioma

Supplementary Figure 1:

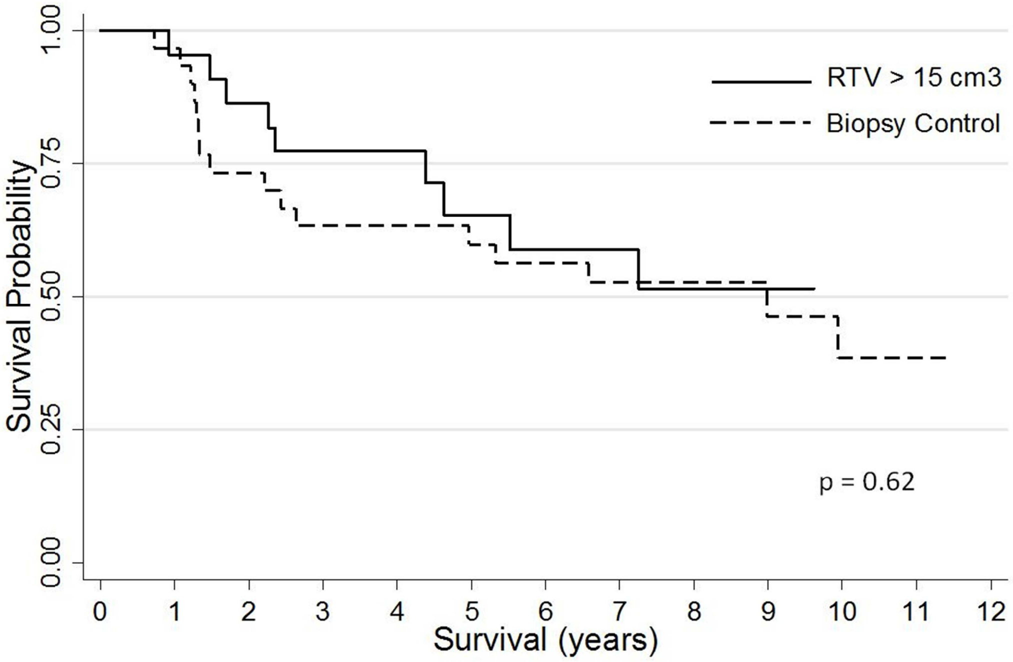

	0	1	2	3	4	5	6	7	8	9	10	11	12
RTV > 15cm^3:	22	22	20	17	14	11	10	9	8	6	0		
Biopsy Control:	30	30	23	20	20	18	17	16	13	8	6	2	0

Numbers-at-Risk

Overall survival of low grade glioma patients with a RTV of more than 15cm3 compared to a matching control group with initial biopsy ("Biopsy Control"). No difference in overall survival was observed between both groups.

Supplementary Figure 2:

	Early RTX		
Histology	no	yes	Total
Oligodendroglioma	15	1	16
Oligoastrocytoma	42	1	43
Astrocytoma	34	22	56
Total	91	24	115
Pearson $\chi 2$	25.0, $p < 0.001$		

Early radiotherapy (within 6 months of diagnosis) was predominantly applied in patients with astrocytoma histology.

Supplementary Table 1:

	Initial Management			Univariate Statistics
	**Biopsy**	**Resection**		
**Patient characteristics**		**Residual Tumor Volume < 15cm^3**	**Residual Tumor Volume > 15cm^3**	
Number of patients	77	27	22	
Year of first diagnosis, (IQR), y	2006 (2005-2008)	2009 (2006-2010)	2007 (2006-2011)	**p= 0.0104**
Sex				χ^2 (2, N=126) = 1.7 p = 0.42
Male	50 (65%)	21 (78%)	16 (73%)	
Female	27 (35%)	6 (22%)	6 (27%)	
Age at diagnosis, median (IQR), y	44 (35-60)	39 (27-45)	42 (36-50)	p = 0.13
Follow-up, median, (IQR), y	4.3 (2.1-8.2)	5.8 (4.2-8.6)	4.7 (2.4-9.4)	p = 0.103
Status				χ^2 (4, N=126) = 13.8 **p = 0.008**
Dead	43 (56%)	4 (15%)	9 (41%)	
Alive	31 (40%)	21 (78%)	12 (55%)	
Lost to follow-up	3 (4%)	2 (74%)	1 (5%)	
Overall survival, median, y	6.7	Not reached	10.6	**p = 0.0025**
5-year survival rate (%)	54%	96%	64%	
10-year survival rate (%)	38%	77%	58%	
Preoperative KPS, mean (SD), %	90 (8.0)	93 (9.9)	90 (6.5)	**p = 0.032**
Charlson comorbidity index, mean (SD)	0.32 (1.1)	0.41 (0.7)	0.32 (0.8)	p = 0.28
Initial symptoms				χ^2 (6, N=126) = 3.2 p = 0.79
None (incidental)	5 (6%)	1 (4%)	1 (5%)	
Seizure	49 (64%)	18 (67%)	16 (7%)	
Headache	10 (13%)	1 (4%)	5 (23%)	
Neurological deficit	23 (30%)	8 (30%)	7 (32%)	
Pignatti score, mean (SD)	1.8 (1.0)	1.4 (1.2)	2.0 (1.3)	p = 0.105
**Tumor Characteristics**				
Histopathology				χ^2 (4, N=126) = 10.1 **p = 0.039**
Astrocytoma WHO°II	44 (57%)	10 (37%)	8 (36%)	
Oligoastrocytoma WHO°II	27 (35%)	9 (33%)	10 (45%)	
Oligodendroglioma WHO°II	6 (8%)	8 (30%)	4 (18%)	
Tumor size				
Maximum diameter, mean, (SD), mm	5.0 (1.9)	4.8 (2.2)	7.0 (1.8)	**p < 0.001**
Tumor volume, mean, (SD), mm^3	54 (53)	41 (36)	117 (59)	**p < 0.001**
Eloquent location (Sawaya-Score)				χ^2 (4, N=126) = 26.5 **p < 0.001**
1	5 (6%)	8 (30%)	0 (0%)	
2	50 (65%)	17 (63%)	8 (36%)	
3	32 (42%)	2 (7%)	14 (64%)	
Bilateral tumor extension	4 (5%)	1 (4%)	2 (9%)	χ^2 (2, N=126) = 0.72 p = 70
Left Hemisphere	45 (58%)	15 (56%)	10 (45%)	χ^2 (2, N=126) = 0.82 p = 0.66
Preoperative contrast enhancement	14 / 73§ (19%)	6 / 24§ (25%)	7 / 17§ (41%)	χ^2 (2, N=) = 4.0 p = 0.14
Location of tumor				χ^2 (8, N=126) = 14.4 p = 0.073
Frontal	29 (38%)	14 (52%)	8 (36%)	
Temporal	10 (13%)	8 (30%)	3 (14%)	
Parietal	7 (9%)	2 (7%)	0 (0%)	
Insula	20 (26%)	2 (7%)	9 (41%)	
Other	11 (14%)	1 (4%)	2 (9%)	
**Surgical Characteristics**				
Number of tumor resections				
0 (Biopsy only)	55 (71%)	-	-	
1	13 (17%)	17 (63%)	8 (4%)	χ^2 (4, N=) = 5.6

2	6 (8%)	9 (33%)	12 (55%)	p = 0.23
>2	3 (4%)	1 (4%)	2 (9%)	
Post operative deficits†	0 (0%)	5 (19%)	2 (9%)	
Post-operative deficits > 6 months	0 (0%	2 (7%)	2 (9%)	
**Adjuvant Therapy**				
	Available:66 (86%)	Available: 27 (%)	Available: 22 (%)	
None	22 (32%)	14 (52%)	9 (41%)	**χ^2 (8, N=) = 16.7 p = 0.033**
Early radiotherapy	22 (32%)	1 (4%)	1 (5%)	
Ever radiotherapy	37 (56%)	11 (41%)	10 (45%)	
Early chemotherapy	19 (29%)	4 (15%)	1 (5%)	
Ever chemotherapy	41 (62%)	11 (41%)	12 (55%)	

§ Number of available pre-operative contrast-enhanced MRI

† Deficits after first intervention (biopsy or 1st surgery), permanent deficit was defined as a new deficit persisting > 6 months

Supplementary Table 2:

	**Biopsy Matching Group**	**Resection Residual Tumor Volume > 15cm^3**	Univariate Statistics
**Patient characteristics**			
Number of patients	30	22	
Year of first diagnosis, mean (SD)	2005 (2005-2007)	2007 (2006-2011)	**0.003**
Sex			p = 0.76
Male	20 (67%)	16 (%)	
Female	10 (33%)	6 (%)	
Age at diagnosis, median (IQR), y	44 (36-54)	42 (36-50)	p = 0.52
Follow-up, median (IQR), y	6.0 (1.5-9.3)	4.7 (2.4-9.4)	p = 0.82
Status			χ^2 (2, N=52) = 1.3 p = 0.53
Dead	17 (57%)	9 (%)	
Alive	12 (40%)	12 (%)	
Lost to follow-up	1 (3%)	1 (%)	
Overall Survival, median, y	9.1	10.6	p = 0.64
5-year survival rate (%)			
10-year survival rate (%)			
Preoperative KPS, mean (SD), %	88 (9.1)	90 (6.5)	p = 0.50
Charlson comorbidity index, mean (SD)	0.17 (53)	0.32 (0.8)	p = 0.41
Initial symptoms			χ^2 (3, N=52) = 3.4 p = 0.34
None (incidental)	0 (0%)	1 (%)	
Seizure	22 (73%)	16 (%)	
Headache	2 (7%)	5 (%)	
Neurological deficit	10 (30%)	7 (%)	
Pignatti score, mean (SD)	2.1 (1.1)	2.0 (1.3)	p = 0.79
**Tumor Characteristics**			
Histopathology			χ^2 (2, N=52) = 2.3 p = 0.31
Astrocytoma WHO°II	16 (53%)	8 (%)	
Oligoastrocytoma WHO°II	12 (40%)	10 (%)	
Oligodendroglioma WHO°II	2 (7%)	4 (%)	
Tumor size			
Maximum diameter, mean, (SD), mm	65 (14)	70 (18)	p = 0.18
Tumor volume, mean, (SD), mm^3	91 (43)	117 (59)	p = 0.10

Eloquent location (Sawaya-Score)			
1	0 (0%)	0 (0%)	χ^2 (1, N=52) = 0.96<br>p = 0.62
2	15 (50%)	8 (%)	
3	15 (50%)	14 (%)	
Bilateral tumor extension	3 (10%)	2 (%)	p = 1.00
Left Hemisphere	16 (53%)	10 (%)	p = 0.77
Preoperative contrast enhancement	9 / 28[§] (32%)	7 / 17[§] (%)	p = 0.19
Location of tumor			χ^2 (4, N=126) = 4.1<br>p = 0.40
Frontal	13 (43%)	8 (%)	
Temporal	1 (3%)	3 (%)	
Parietal	1 (3%)	0 (%)	
Insula	9 (30%)	9 (%)	
Other	6 (20%)	2 (%)	
Anaplastic transformation[#]	32 / 42 (76%)	21 / 25 (84%)	
			p = 0.54
**Surgical Characteristics**			
Number of tumor resections			
0 (Biopsy only)	22 (73%)	-	
1	4 (13%)	8 (%)	χ^2 (2, N=30) = 0.68<br>p = 0.71
2	3 (10%)	12 (%)	
>2	1 (3%)	2 (%)	
Post operative deficits[†]	0 (0%)	2 (9%)	p = 0.19
Post-operative deficits > 6 months	0 (0%)	2 (9%)	p = 0.19
**Adjuvant Therapy**	Available: 27 (%)	Available: 22 (%)	
None	7 (26%)	9 (41%)	χ^2 (4, N=49) = 8.7<br>p = 0.07
Early radiotherapy	9 (33%)	1 (5%)	
Ever radiotherapy	14 (52%)	10 (45%)	
Early chemotherapy	8 (30%)	1 (5%)	
Ever chemotherapy	17 (63%)	12 (55%)	

[§] Number of available pre-operative contrast-enhanced MRI

[†] Deficits after first intervention (biopsy or 1st surgery), permanent deficit was defined as a new deficit persisting > 6 months
